# Pre-treatment fertility preservation and post-treatment reproduction in long-term survivors of adolescent and young adult (AYA) cancer

**DOI:** 10.1007/s11764-024-01538-x

**Published:** 2024-02-06

**Authors:** Vicky Lehmann, Carla Vlooswijk, Winette T. A. van der Graaf, Rhodé Bijlsma, Suzanne E. J. Kaal, Jan Martijn Kerst, Jacqueline M. Tromp, Monique E. M. M. Bos, Tom van der Hulle, Roy I. Lalisang, Janine Nuver, Mathilde C. M. Kouwenhoven, Christianne A. R. Lok, Catharina C. M. Beerendonk, Marij Dinkelman-Smit, Olga Husson

**Affiliations:** 1 Department of Medical Psychology, Amsterdam University Medical Center/University of Amsterdam, Meibergdreef 9, 1105 AZ Amsterdam, The Netherlands; 2 Cancer Center Amsterdam (CCA), Amsterdam, The Netherlands; 3 Department of Research and Development, Netherlands Comprehensive Cancer Organisation (IKNL), Utrecht, The Netherlands; 4 Department of Medical Oncology, Netherlands Cancer Institute, Amsterdam, The Netherlands; 5 Department of Medical Oncology, ErasmusMC Cancer Institute, Erasmus University Medical Center, Rotterdam, The Netherlands; 6 Department of Medical Oncology, UMC Utrecht Cancer Center, Utrecht, The Netherlands; 7 Department of Medical Oncology, Radboud University Medical Center, Nijmegen, The Netherlands; 8 Department of Medical Oncology, Netherlands Cancer Institute-Antoni Van Leeuwenhoek, Amsterdam, The Netherlands; 9 Department of Medical Oncology, Amsterdam University Medical Center/University of Amsterdam, Amsterdam, The Netherlands; 10 Department of Medical Oncology, Leiden University Medical Center, Leiden, The Netherlands; 11 Department of Internal Medicine, GROW-School of Oncology and Reproduction, Maastricht UMC, Maastricht, The Netherlands; 12 Department of Medical Oncology, University Medical Center Groningen, Groningen, The Netherlands; 13 Department of Neurology, Amsterdam University Medical Centers/University of Amsterdam, Amsterdam, The Netherlands; 14 Department of Gynecologic Oncology, Center Gynaecologic Oncology Amsterdam, Antoni Van Leeuwenhoek, Amsterdam, The Netherlands; 15 Department of Obstetrics and Gynecology, Radboud University Medical Center, Nijmegen, The Netherlands; 16 Department of Urology, ErasmusMC Cancer Institute, Erasmus University Medical Center, Rotterdam, The Netherlands

**Keywords:** Adolescence and young adulthood (AYA), Reproductive goals, Fertility preservation, Assisted reproductive technologies (ART), Oncology

## Abstract

**Purpose:**

To describe recall of fertility-related consultations and cryopreservation and to examine reproductive goals and reproduction post-treatment in long-term survivors of adolescent and young adult (AYA) (age, 18–39 years) cancer.

**Methods:**

This study included *n* = 1457 male and *n* = 2112 female long-term survivors (*M*_age_ = 43–45 years; 5–22 years from diagnosis) who provided self-report. Clinical data were supplied by the Netherlands Cancer Registry.

**Results:**

Most male survivors (72.7%) recalled fertility-related consultations and 22.6% completed sperm cryopreservation. Younger age (OR = 2.8; 95%CI [2.2–3.6]), not having children (OR = 5.0; 95%CI [3.2–7.7]), testicular cancer or lymphoma/leukemia (OR = 2.8/2.5 relative to “others”), and more intense treatments (OR = 1.5; 95%CI [1.1–2.0]) were associated with higher cryopreservation rates. Time since diagnosis had no effect. Of men who cryopreserved, 12.1% utilized assisted reproductive technologies (ART). Most men (88.5%) felt their diagnosis did not affect their reproductive goals, but 7.6% wanted no (additional) children due to cancer. Half of female survivors (55.4%; *n* = 1171) recalled fertility-related consultations. Rates of cryopreservation were very low (3.6%), but increased after 2013 when oocyte cryopreservation became non-experimental. Of women who cryopreserved, 13.2% successfully utilized ART. Most women (74.8%) experienced no effects of cancer on reproductive goals, but 17.8% wanted no (additional) children due to cancer.

**Conclusions:**

Cryopreservation in men varied by patient/clinical factors and was very low in women, but data of more recently treated females are needed. Utilizing cryopreserved material through ART was rare, which questions its cost-effectiveness, but it may enhance survivors’ well-being.

**Implications for Cancer Survivors:**

The extent to which cryopreservation positively affects survivors’ well-being remains to be tested. Moreover, effects of cancer on reproductive goals require further attention, especially in women who refrain from having children due to cancer.

**Supplementary Information:**

The online version contains supplementary material available at 10.1007/s11764-024-01538-x.

## Introduction

Annually, 3800 adolescents and young adults (AYAs) (age 18–39 years) are diagnosed with cancer in the Netherlands [1, 2]. Many receive gonadotoxic therapies (e.g., fertility-impairing surgery, alkylating chemotherapy, radiation), which put them at risk for temporary or permanent gonadal dysfunction: Depending on age, diagnosis, and treatment, infertility rates in survivors range between 20 and 60%, or above 90% for those receiving stem cell transplants [3–8]. Consequently, survivors can experience distress, depression, anxiety, and relationship difficulties if they encounter fertility problems after treatment [9–16].

To mitigate such negative effects, clinical guidelines [7, 17–20] and patient organizations [21, 22] recommend that all cancer patients of reproductive age should be informed about infertility risks before cancer treatment and be offered fertility preservation, if possible. For decades, the preferred method for fertility preservation in men is sperm cryopreservation. For women, embryo cryopreservation was available, but only for those with a male partner. In 2013, oocyte cryopreservation became available as another non-experimental option for women [20]. If utilized, these preservation methods provide survivors with the option to use assisted reproductive technologies (ART) in survivorship. Additional options to possibly protect ovarian function during treatment may include shielding reproductive organs from radiation or suppressing women’s ovarian function through gonadotropin-releasing hormone (GnRH) agonists [17].

Counseling about infertility risks and referrals to fertility specialists can be challenging: Patients may struggle to understand the complexity of information and procedures, while potentially being overwhelmed by their diagnosis or feeling uncertain about their reproductive goals [23–29]. Additional barriers to cryopreservation include lack of provider knowledge, inadequate hospital referral systems, or advanced disease which require urgent initiation of cancer treatment [28, 30–39]. Irrespective of whether cryopreservation is possible, most survivors highly appreciate having received fertility-related information at diagnosis as they can better prepare for the future [40–42]. Accordingly, survivors can experience despair if they missed or do not recall such conversations [43–45]. Recall of fertility-related counseling does not indicate whether patients were at risk for infertility and eligible for cryopreservation, but such conversations are important for survivors’ quality of life [45, 46] and having a sense of control. Moreover, utilization of cryopreserved material through ART among survivors remains low [47–51], often due to spontaneous pregnancies, but whether attitudes toward having children also change throughout survivorship remains unclear.

First, this study will quantify rates of (a) recalling fertility-related consultations, (b) attempts, and (c) completion of cryopreservation in AYA cancer survivors. We will evaluate whether cryopreservation differs by patient-related/clinical factors in male survivors given that standard cryopreservation was available for them at diagnosis. Second, we will examine whether male and female survivors had children following treatment and how they were conceived. All information will provide a comprehensive overview of cryopreservation, fertility, and reproduction for AYA oncology and survivorship care.

## Methods

### Procedures

Presented data are part of the SURVAYA study [52] among long-term SURVivors of AYA cancer (trial registration: NCT05379387). Eligible survivors were identified through the Netherlands Cancer Registry (NCR), if they were diagnosed with any type of cancer between ages 18 and 39 years in 1999–2015 and were long-term survivors (i.e., ≥ 5 years post-diagnosis).

Between 2019 and 2021, survivors were mailed an invitation package through the PROFILES registry (Patient-Reported Outcomes Following Initial treatment and Long-term Evaluation of Survivorship [53]), which is a data management system to collect patient-reported outcomes and to link clinical data from the NCR. All participants provided written informed consent to participate in this study. Detailed study procedures, along with recruitment strategies and participation rates, were published previously [52]. The institutional review board of the host institute approved this study (NKI-IRBd18122) and participating hospitals provided local approval. All procedures were in accordance with the Declaration of Helsinki.

### Measures

The survey included various multiple-choice questions (see Appendix-A) to assess whether survivors (a) recalled fertility-related *consultations* (yes/no) and whether they (b) unsuccessfully *attempted* or (c) *completed* cryopreservation (i.e., freezing sperm/oocytes/embryo’s/gonadal tissue; Note: fertility-sparring options, like radiation shielding, are reported separately).

Survivors reported whether their *reproductive goals* were directly affected by cancer (i.e., no effect/wanting/not wanting to have (additional) children due to cancer) and whether the treatment had affected their *fertility* (infertile/impaired/at risk, but not formally tested; Appendix-A). *Parental status* (having biological children/not) at diagnosis and post-treatment was assessed. If applicable, survivors reported how they conceived children post-treatment: naturally or through ART (including intra-uterine insemination [IUI], in vitro fertilization [IVF], or intracytoplasmic sperm injection [ICSI]; Appendix-A).

Clinical data from the NCR included age at diagnosis, type of diagnosis, stage, year of diagnosis, and treatment. Treatment information was limited to whether patients received surgery, chemotherapy, or radiation (with/without stem cell transplants [SCT]). This did not allow to differentiate between various chemotherapies, dosage, radiation fields, or surgeries to assess gonadotoxicity. However, we used these primary treatment modalities to code generic *treatment intensity*, based on the premise that more intense treatment regimens were more toxic (overall and for gonads). Survivors were categorized as having received more intense treatments if they got (1) combined chemotherapy and radiation or (2) combined surgery, chemotherapy, and radiation (with/without SCT).

### Statistical analyses

Descriptive statistics will provide rates of (a) recall of fertility-related consultations, (b) attempts, and (c) completion of cryopreservation. For male survivors, determinants of cryopreservation (yes/no) were tested: For continuous factors (age, age at diagnosis, years since diagnosis), *t*-tests were used accompanied by calculating Hedge’s *g* effect sizes to estimate the magnitude of differences. Hedge’s *g* is interpreted like Cohen’s *d* (*g* ≥ 0.2 is small, ≥ 0.5 moderate, ≥ 0.8 large), but uses a correction to prevent overestimation [54]. For categorical factors (parental status, type of diagnosis, stage, treatment intensity), logistic or binomial regression analyses were performed providing odds ratios (OR) and accompanying 95% confidence intervals (CIs). Each factor was tested separately to assess its unique contribution. For male and female survivors, descriptive statistics of reproductive goals, reproduction, and utilizing ART post-treatment were calculated.

## Results

### Men

Of *N* = 4631 eligible male survivors, *N* = 1549 participated (33.4%) and *N* = 1457 provided fertility-related data to be included here. Participants were 43.3 years old and most were highly educated (55.7%). Their age at diagnosis was 30.1 years, and most had been diagnosed with testicular cancer (43.0%). Less than half of men were diagnosed with stage I disease (37.3%). Treatment intensity was categorized as high in 17.8% of survivors (Table 1; and see clinical data stratified by diagnosis in Appendix-1).

**Table 1:** Sociodemographic information and clinical data in male and female survivors

	Male survivors	Male survivors		Female survivors
	(*N* = 1457)	With FP	Without FP	(*N* = 2112)
		*n* = 330	*n* = 1127	
	*M* (SD), range	*M* (SD)	*M* (SD)	*M* (SD), range
Age at diagnosis	30.1 (6.2), 18–39	27.6 (5.4)	30.9 (6.2)	32.5 (5.6), 18–39
Age at study	43.3 (7.7), 24–60	40.5 (7.1)	44.1 (7.6)	45.3 (7.2), 23–61
Years since diagnosis	12.6 (4.5), 5–22	12.4 (4.5)	12.7 (4.5)	12.3 (4.5), 5–22
	*n (%)*	*n (%)*	*n (%)*	*n (%)*
5 + years (5–9 years)	484 (33.2%)	110 (33.3%)	374 (33.2%)	731 (34.6%)
10 + years (10–14 years)	495 (34.0%)	117 (35.5%)	378 (33.5%)	758 (35.9%)
15 + years (15–22 years)	478 (32.8%)	103 (31.2%)	375 (33.3%)	623 (29.5%)
Relationship status				
Single	247 (17.0%)	60 (18.2%)	187 (16.6%)	342 (16.2%)
Partnered	1206 (82.8%)	269 (81.5%)	937 (83.1%)	1768 (83.7%)
Missing	4 (0.3%)	1 (0.3%)	3 (0.3%)	2 (0.1%)
Level of education				
Low *(no/secondary education)*	11 (0.8%)	1 (0.3%)	10 (0.9%)	6 (0.3%)
Middle* (secondary vocational training)*	630 (43.2%)	118 (35.8%)	512 (45.4%)	873 (41.3%)
High *(college/university degree)*	812 (55.7%)	211 (63.9%)	601 (53.3%)	1230 (58.2%)
Missing	4 (0.3%)	-	4 (0.4%)	3 (0.1%)
Type of diagnosis				
Testicular cancer	626 (43.0%)	177 (53.6%)	449 (39.8%)	-
Breast cancer	-	-	-	812 (38.4%)
Lymphoma/leukemia	356 (24.4%)	94 (28.5%)	262 (23.2%)	315 (14.9%)
Cervical cancer	-	-	-	306 (14.5%)
Other types, including:	475 (32.6%)	59 (17.9%)	416 (36.9%)	679 (32.1%)
Melanoma	82 (5.6%)	-	82 (7.3%)	173 (8.2%)
Bone/soft tissue sarcomas	77 (5.3%)	16 (4.8%)	61 (5.4%)	79 (3.7%)
CNS malignancies	69 (4.7%)	13 (3.9%)	56 (5.0%)	63 (3.0%)
Digestive tract/colon/rectal cancer	64 (4.4%)	7 (2.1%)	57 (5.1%)	38 (1.8%)
Head and neck cancer	62 (4.3%)	5 (1.5%)	57(5.1%)	46 (2.2%)
Thyroid cancer	56 (3.8%)	9 (2.7%)	47 (4.2%)	162 (7.7%)
Cancer of other female genitalia	-	-	-	71 (3.4%)
Others with *n* < 50 in whole sample	65 (4.5%)	9 (2.7%)	56 (5.0%)	47 (2.2%)
Disease stage				
I	544 (37.3%)	115 (34.8%)	429 (38.1%)	992 (47.0)
II	328 (22.5%)	94 (28.5%)	234 (20.8%)	606 (28.7%)
III	274 (18.8%)	66 (20.0%)	208 (18.5%)	241 (11.4%)
IV	89 (6.1%)	13 (3.9%)	76 (6.7%)	70 (3.3%)
Unknown	222 (15.2%)	42 (12.7%)	180 (16.0%)	203 (9.6%)
Primary treatment modalities				
Surgery	302 (20.7%)	32 (9.7%)	270 (24.0%)	562 (26.6%)
Chemotherapy	219 (15.0%)	55 (16.7%)	164 (14.6%)	189 (8.9%)
Radiation	53 (3.6%)	8 (2.4%)	45 (4.0%)	40 (1.9%)
Surgery + chemotherapy	383 (26.3%)	117 (35.5%)	266 (23.6%)	206 (9.8%)
Surgery + radiation	270 (18.5%)	52 (15.8%)	218 (19.3%)	304 (14.4%)
Chemotherapy + radiation*	169 (11.6%)	56 (17.0%)	113 (10.0%)	209 (9.9%)
Surgery + chemotherapy + radiation*	40 (2.7%)	8 (2.4%)	32 (2.8%)	586 (27.7%)
Other/missing	21 (1.5%)	2 (0.6%)	19 (1.7%)	16 (0.8%)
Stem cell transplant (SCT)*	68 (4.7%)	13 (3.9%)	55 (4.9%)	63 (3.0%)
High treatment intensity	260 (17.8%)	75 (22.7%)	185 (16.4%)	838 (39.7%)

^*^Included in high treatment intensity

### Cryopreservation

Almost three-quarters of male survivors (72.7%) recalled fertility-related consultations before cancer treatment. Less than one quarter (22.6%, *n* = 330) completed sperm cryopreservation at diagnosis (96.4% froze fresh semen, *n* = 318/330). Another 4.0% (*n* = 58) reported unsuccessful attempts to cryopreserve (Table 2/ Appendix-1). Few men (*n* = 18, 1.2%) reported additional radiation shielding.

**Table 2:** Outcomes in participating male and female survivors

	Male survivors	Male survivors		Female survivors
	(*N* = 1457)	With FP	Without FP	(*N* = 2112)
		*n* = 330	*n* = 1127	
	*n* (%)	*n* (%)	*n* (%)	*n* (%)
Impact cancer on reproductive goals				
No impact	1289 (88.5%)	292 (88.5%)	997 (88.5%)	1579 (74.8%)
Wanted (additional) children	54 (3.7%)	20 (6.1%)	34 (3.0%)	142 (6.7%)
Wanted no children	111 (7.6%)	18 (5.5%)	93 (8.3%)	376 (17.8%)
Missing	3 (0.2%)	-	3 (0.3%)	15 (0.7%)
Parental status at diagnosis^†^				
No children	655 (45.0%)	167 (50.5%)	488 (43.3%)	786 (36.4%)
Had children	406 (27.9%)	26 (7.9%)	380 (33.7%)	956 (45.3%)
Missing/timing unknown^a^	396 (27.2%)	137 (41.5%)	259 (23.0%)	388 (18.4%)
Parental status at study				
No children	500 (34.3%)	120 (36.4%)	380 (33.7%)	641 (30.4%)
*Still trying post-treatment*	*166/500 (33.2%)*	*69/120 (57.5%)*	*97/380 (25.5%)*	*110/641 (18.5%)*
*No desire to have children*^h^	*209/500 (41.8%)*	*36/120 (30.0%)*	*173/380 (45.5%)*	*285/641 (44.5%)*
Had children	957 (65.7%)	210 (63.6%)	747 (66.3%)	1469 (69.6%)
*Conceived post-treatment*^g^	*510/957 (53.3%)*	*188/210 (89.5%)*	*322/747 (43.1%)*	*417/1469 (28.4%)*
Ongoing pregnancy	6 (0.4%)	1 (0.3%)	5 (0.4%)	12 (0.6%)
Ways of conceiving post-treatment^b^				
Naturally	389/510 (76.3%)	142/188 (75.5%)	247/322(76.7%)	373/417 (89.5%)
IUI	56/510 (11.0%)	19/188 (10.1%)	37/322 (11.5%)	4/417 (1.0%)
ICSI	33/510 (6.5%)	12/188 (6.4%)	21/322 (6.5%)	29/417 (7.0%)^e^
IVF	19/510 (3.7%)	9/188 (4.8%)	10/322 (3.1%)	
Naturally and ART (multiple pregnancies)	11/510 (2.2%)	5/188 (2.7%)	6/322 (1.9%)	-
Hormones	1/510 (0.2%)	1/188 (0.5%)	-	11/417 (2.6%)
Missing	1/510 (0.2%)	-	1/322 (0.3%)	-
Recall of fertility consult				
	1059 (72.7%)	317 (96.1%)	742 (65.8%)	1171 (55.4%)
Cryopreservation^c^				
No attempt	987 (67.7%)	-	-	1917 (90.8%)
Completed cryopreservation	330 (22.6%)^d^	-	-	76/2112 (3.6%)^f^
Attempted, but not completed	58 (4.0%)	-	-	85/2112 (4.0%)
Missing	82 (5.7%)	-	-	34 (1.6%)
Beliefs treatment affected fertility^i^	591 (40.6%)	153 (46.4%)	438 (38.9%)	1067 (50.5%)
These effects were:				*n* = 555, aged < 40
Infertile/ sterile	205/591 (34.7%)	41 (26.8%)	164 (37.4%)	95/555 (17.1%)
Impaired/subfertile	144/591 (24.4%)	49 (32.0%)	95 (21.7%)	83/555 (15.0%)
(Likely) impaired, but not tested	116/591 (19.6%)	30 (19.6%)	86 (19.6%)	21/555 (3.8%)
(Likely) fertile	27/591 (4.6%)	11 (7.2%)	16 (3.7%)	19/555 (3.4%)
Missing	99/591 (16.8%)	22 (13.7%)	77 (17.6%)	81/55 (14.6%)

^**†**^*n* = 23 women were pregnant at diagnosis

^a^Unclear if participants had children before or after treatment

^b^Male survivors: another *n* = 5 had children by utilizing donor sperm, *n* = 52 tried ART but did not work; female survivors: another *n* = 11 egg donation, *n* = 8 surrogate, *n* = 62 tried ART but did not work

^c^*n* = 18 (male) and *n* = 11 (female) got additional shielding from radiation as a means to spare fertility

^d^Cryopreservation of fresh semen *n* = 318/330 (96.4%), note that information about possible TESE or PESA was ambiguous and therefore not reported

^e^Includes ICSI and IVF

^f^Cryopreserved oocytes (*n* = 34), embryos (*n* = 38), or ovarian tissue (*n* = 12; eight women indicated two options)

^g^Number of survivors for whom paternity/maternity could be confirmed as being post-treatment

^h^No desire to have children due to cancer in men, 15.8% (*n* = 33/209), and in women, 23.5% (*n* = 67/205)

^i^See Appendix-A for categorizations

### Determinants

Men who completed cryopreservation were younger at diagnosis (*M* = 27.6 vs. 30.9 years, *t*(1455) = 8.64, *p* < 0.001; *g* = 0.54; Table 1). Accordingly, men diagnosed in their 20 s were almost three times more likely to cryopreserve than men in their 30 s (OR = 2.8; 95%CI [2.2–3.6]; Table 3). Men’s level of education appeared influential, but the subgroup of lower-educated men was too small for formal testing (*n* = 11). Men without children at diagnosis were five times more likely to cryopreserve than those who already had children (OR = 5.0; 95%CI [3.2–7.7]; Table 3).

**Table 3:** Logistic regression analyses on completion of sperm cryopreservation in male survivors

Factor	Cryopreservation	No cryopreservation	OR (95%CI)	Model	Nagelkerke *R*^2^
Model 1: age at diagnosis					
18–29 years	206 (32.8%)	422 (67.2%)	2.8 (2.2–3.6)*	*χ*^*2*^(1) = 64.60, *p* < .001	.066
30–39 years	124 (15.0%)	705 (85.0%)	1		
Model 2: parental status at diagnosis					
No children	167 (25.5%)	488 (74.5%)	5.0 (3.2–7.7)*	*χ*^*2*^(1) = 69.48 *p* < .001	.103
Had children	26 (6.4%)	380 (93.6%)	1		
Model 3: type of diagnosis (‘other’ as reference)					
Lymphoma/leukemia	94 (26.4%)	262 (73.6%)	2.5 (1.8–3.6)*	*χ*^*2*^(2) = 45.93; *p* < .001	.047
Testicular	177 (28.3%)	449 (71.7%)	2.8 (2.0–3.8)*		
Other	59 (12.4%)	416 (87.6%)	1		
Model 3a: type of diagnosis (lymphoma/leukemia as reference)					
Lymphoma/leukemia	94 (26.4%)	262 (73.6%)	1	*χ*^*2*^(2) = 42.93; *p* < .001	.047
Testicular	177 (28.3%)	449 (71.7%)	1.1 (0.8–1.5)		
Other	59 (12.4%)	416 (87.6%)	0.4 (0.3–0.6)*		
Model 4: disease stage (stage IV as reference)					
I	115 (21.1%)	429 (78.9%)	1.6 (0.8–2.9)	*χ*^*2*^(3) = 10.75; *p* = .013	.013
II	94 (28.7%)	234 (71.3%)	2.3 (1.2–4.4)*		
III	66 (24.1%)	208 (75.9%)	1.9 (0.9–3.6)		
IV	13 (14.6%)	76 (85.4%)	1		
Model 4a: disease stage (stage I as reference)					
I	115 (21.1%)	429 (78.9%)	1	*χ*^*2*^(3) = 10.75; *p* = .013	.013
II	94 (28.7%)	234 (71.3%)	1.5 (1.1–2.1)*		
III	66 (24.1%)	208 (75.9%)	1.2 (0.8–1.7)		
IV	13 (14.6%)	76 (85.4%)	0.2 (0.3–1.2)		
Model 5: treatment intensity					
(More) intense^†^	75 (28.8%)	185 (71.2%)	1.5 (1.1–2.0)*	*χ*^*2*^(1) = 6.64; *p* = .010	.007
Less intense/invasive	255 (21.3%)	942 (78.7%)	1		

^*^Significant at *p* < .05

^†^Combination of chemotherapy and radiation, or combination of all primary treatment modalities, and/or a SCT

No difference was seen between testicular cancer versus lymphoma/leukemia survivors (OR = 1.1; 95%CI [0.8–1.5]), but both were almost three times more likely to cryopreserve than survivors of “other” cancers (OR = 2.8/2.5, respectively). This equates to 28.3% of testicular cancer and 26.4% lymphoma/leukemia survivors who cryopreserved, versus 12.4% of men with “other” diagnoses (Table 2 and 3/ Appendix-1).

Cryopreservation by disease stage showed the highest rates in men with stage II (28.7%) and III disease (24.1%), while it was less common in men with stage IV disease (14.6%). Accordingly, men with stage II disease were more than two times more likely to cryopreserve than men with stage IV disease (OR = 2.3; 95%CI [1.2–4.4]) and 1.5 times more likely than men with stage I disease (OR = 1.5; 95%CI [1.1–2.1]), while other groups were comparable (Table 3). Yet, rates were similar across stages if stratified by diagnostic groups (Appendix-1).

Men who had received more intense cancer treatments were more likely to cryopreserve before treatment (OR = 1.5; 95%CI [1.1–2.0]; Table 3). Specifically, of men who cryopreserved, 35.5% received surgery combined with chemotherapy (Table 1/Appendix-1).

Years since diagnosis did not differ between men who cryopreserved and those who did not (*M* = 12.4 vs. 12.7 years; *t*(1455) = 1.06, *p* = 0.146, *g* = 0.07; Table 1). Accordingly, cryopreservation rates were similar (*χ*^2^(2) = 0.61; *p* = 0.738) for survivors diagnosed 5 + , 10 + , or 15 + years ago (Table 1). There were also no trends in cryopreservation rates over time in each diagnostic group (Appendix-1-Figure).

### Men’s reproduction

Following treatment, 40.6% of men (*n* = 591) indicated that cancer treatment had affected their fertility. They specified being infertile/sterile (34.7%, *n* = 205/591), having impaired fertility (24.4%, *n* = 144/591), or being told they were at risk, but not tested (19.6%, *n* = 116/591; Table 2). Notably, 20.0% of reportedly infertile men (*n* = 41/205) completed cryopreservation at diagnosis.

Most men (88.5%, *n* = 1289) felt their cancer did not affect their reproductive goals, whereas 7.6% did not want to have any (additional) children due to cancer. Most survivors had children (*n* = 957; 65.7%), of which 53.3% (*n* = 510/957) were conceived following treatment. This included 76.3% (*n* = 389/510) who conceived naturally, 21.2% (*n* = 108/510) conceived through ARTs (*n* = 56 IUI, *n* = 33 ICSI, *n* = 19 IVF), and another *n* = 11 conceived several children both naturally and through ART (Table 2). Of all men, *n* = 52 (3.4%) had unsuccessfully tried ART. Of men without children (*n* = 500; 34.3%), 33.2% (*n* = 166/500) were still trying to sire a pregnancy, whereas 41.8% (*n* = 209/500) expressed no desire to have any children, which was due to cancer in 15.8% of these men (*n* = 33/209; see Table 2).

Of *n* = 330 men who cryopreserved sperm, *n* = 188 (57.0%) had children post-treatment. This included 75.5% (*n* = 142/188) who conceived naturally (which equals 43.0% of all men who cryopreserved, *n* = 142/330), whereas 21.3% (*n* = 40/188) conceived through ART (equals 12.1% of all men who cryopreserved, *n* = 40/330), including IUI (*n* = 19), ICSI (*n* = 120), and IVF (*n* = 9) (Table 2).

### Women

Of *N* = 6665 eligible female survivors, *N* = 2461 participated (36.9%) and *N* = 2112 provided relevant data. Women were 45.3 years old and most were highly educated (58.2%; Table 1). They were 32.5 years old at diagnosis, which was most frequently breast cancer (38.4%). Many women had been diagnosed with stage I disease (47.0%), and many received intense treatments (39.7%; Table 1; see treatment-related data stratified by diagnosis in Appendix-2).

### Cryopreservation

About half of women (55.4%; *n* = 1171) recalled fertility-related consultations before cancer treatment. A minority of 3.6% (*n* = 76/2112) cryopreserved oocytes (*n* = 34), embryos (*n* = 38), or ovarian tissue (*n* = 12; eight women indicated two options). Few women reported (additional) ovarian transposition (*n* = 11) or suppression (*n* = 25). Another 4.0% (*n* = 85/2112) reported unsuccessful attempts to cryopreserve (Table 2). Among women diagnosed after 2013, a higher proportion (10.0%; *n* = 24/239) completed cryopreservation before treatment (particularly those with breast cancer; Appendix-2).

### Women’s reproduction

Following treatment, 50.5% of women (*n* = 1067/2112) believed that cancer treatment had affected their fertility. Yet, more women indicated fertility problems in open-ended questions, but the timeline of symptom onset was often unclear (i.e., premature vs. natural menopause). Women younger than 40 years at the study (*n* = 555) often believed that cancer had no effect on their fertility (46.1%; *n* = 256/555), whereas 17.1% (*n* = 95/555) reported being infertile (Table 2/Appendix-3). This included *n* = 32 with diagnosed premature ovarian insufficiency (POI), *n* = 18 with hysterectomies, *n* = 5 with bilateral oophorectomy, and *n* = 40 did not specify the nature of their infertility. Similarly, women aged 40–45 at study participation (*n* = 549) often believed that cancer had no effect on their fertility (39.2%; *n* = 215/549), whereas 26.6% reported being infertile (*n* = 146/549; including *n* = 38 POI, *n* = 37 hysterectomies, *n* = 25 bilateral oophorectomy, *n* = 46 did not specify; Appendix-3).

Most women (74.8%) felt that cancer did not affect their reproductive goals, but 17.8% indicated not wanting to have (additional) children due to cancer (Table 2). At study, most female survivors (69.6%; *n* = 1469) had children, of which only 28.4% (*n* = 417; equals 19.7% of the whole sample) had conceived post-treatment. Most women conceived naturally (89.4%; *n* = 373/471) and 7.9% utilized ART (*n* = 33/417; Table 2). Of women who were without children (641; 30.4%), 18.5% (*n* = 110/641) were still trying to conceive, whereas 44.5% (*n* = 285/641) expressed no desire to have any children, which was due to cancer in 23.5% of these women (*n* = 67/285; Table 2).

Of *n* = 76 women who completed cryopreservation, 25% conceived naturally (*n* = 19/76). Another *n* = 17 (22.4%) utilized ART, which resulted in having a child in *n* = 10/17 (58.8%) women, but was unsuccessful for *n* = 7/17 (41.2%).

## Discussion

This study of AYA cancer survivors demonstrated high rates of recall of fertility-related consultations in men and lower rates in women, while relatively few men and fewer women completed cryopreservation before starting cancer treatment. Cryopreservation rates improved for women after the introduction of standard oocyte cryopreservation. However, inequalities in opportunities to cryopreserve between men and women remain, as options for women are more complex and time-consuming [55–57], but ovarian tissue freezing (OTC) may be another less time-consuming option for currently treated women. Many male and female survivors became parents through natural conception after treatment, while utilizing cryopreserved material through ART was limited, questioning its cost-effectiveness and adequacy of fertility-related counseling at diagnosis. Nevertheless, fertility-related consultations and options for future ART may provide cancer patients and survivors with hope and a positive outlook on life [45, 46].

### Male survivors

Most men recalled fertility-related discussions with providers even long after treatment, which corroborates research in Swedish AYA cancer survivors [55, 58]. It reassures that providers pay attention to the reproductive health of male AYA cancer patients. Notably, cryopreservation is covered by health insurance in the Netherlands, which represents a crucial barrier to fertility preservation in other countries [27]. Despite being informed, 22.6% completed and 4.0% attempted semen cryopreservation before cancer treatment. Whether unsuccessful attempts were due to azoospermia or inability to produce any semen was not included in this study. Previous research showed that men with testicular cancer produce semen of lower quality [59] and testicular sperm extraction (TESE) can routinely be offered to post-pubertal males with azoospermia at cancer diagnosis in the Netherlands since 2015. This likely contributes to higher cryopreservation rates in more recently treated samples.

Cryopreservation was higher in men with testicular cancer or lymphoma/leukemia (~ 28%) relative to other diagnoses, but still lower than previously reported rates in men with testicular (49%, [56]) or mixed types of cancer (54%, [55]). Underlying reasons remain unclear, but other countries may use different referral protocols. Before 2015, testicular cancer patients in the Netherlands were typically only referred to fertility services, if their intended treatment included chemotherapy.

Consistent with previous research [60], younger men were more likely to complete cryopreservation, which is likely intertwined with not having children at diagnosis [56], as also identified in this study. Men diagnosed at stage IV were least likely to cryopreserve, potentially due to their disease progression/poor health and prioritizing cancer treatment. Men who received more intense treatments were more likely to cryopreserve. Thus, providers likely anticipated greater infertility risks based on impending treatments and referred patients to fertility services accordingly.

Despite increasing attention to oncofertility in research and care [61], rates of cryopreservation remained constant over time, and almost half of participating men reported negative treatment-related effects on fertility. Men who were infertile/sterile post-treatment had completed cryopreservation at similar rates than the rest of the sample (20 vs. 22%). In other words, some missed opportunities to cryopreserve leaving them infertile and without options for biological fatherhood after treatment. It remains to be tested whether providers incorrectly evaluated infertility risk or whether patients themselves rejected cryopreservation. At the same time, it also underlines the complexity of predicting patients’ future reproductive health [5] and need for adequate counseling [7, 17–20] at diagnosis and follow-up care.

### Female survivors

Half of all women received fertility-related counseling, which likely focused on risks rather than cryopreservation given the era of diagnosis (1999–2015). Accordingly, very few women completed cryopreservation, which tripled after 2013 due to the introduction of oocyte cryopreservation as a non-experimental option, and some even completed OTC at that time. Similar low cryopreservation rates of women treated in these times have been described previously [62, 63], and fertility-related counseling in female cancer patients continues to be suboptimal [64]. Additional research in more recently treated AYA women is needed.

Interestingly, almost half of reproductive-age women believed that their cancer treatment did not affect their fertility. We could not objectively assess gonadal functioning, but we question this rather optimistic outlook: 40% received intense treatments, which could have put them at risk for fertility problems. Previous research showed that survivors’ perceived infertility risks do not necessarily align with actual risks and gonadal functioning later in life [65]. Thus, counseling these women about reproductive health throughout survivorship is crucial.

### Reproductive goals

After treatment, survivors sometimes reconsider their life goals [11, 13, 66], and this study showed that almost 20% of female, but only 8% of male survivors wanted no (additional) children due to their cancer experience. This corroborates qualitative studies where mostly female survivors are concerned about relapses and their ability to have a healthy pregnancy [67–71]. Inherently, the physical demands of a pregnancy are of greater concern to female survivors, and they are less likely to reproduce than male AYA survivors [72]. Fertility problems can also be problematic for male survivors when dating women, as these women potentially face intensive ART procedures or a life without children, causing worries and relationship problems as seen in our clinical practice. Accordingly, male survivors are less likely to become fathers than peers [73]. Both male and female survivors may struggle with romantic relationships and feelings of femininity/masculinity due to fertility problems [11, 40, 74, 75], which needs attention in follow-up care.

Few male and female participants who had completed cryopreservation also conceived through ART (12/13%), questioning the cost-effectivity of cryopreservation [76]. Similar low uptake of cryopreserved material (5–11%) was described previously [47–51], and more research is needed into the decision-making and pathways of (in)voluntary childlessness. Nevertheless, the possibility of ART may foster hope or reassurance for later in life [77], irrespective of uptake.

### Limitations

Recall bias may be present given the long time since diagnosis. However, relatively high rates of recalled counseling (and similar cryopreservation rates over time in men) are reassuring that recall bias is likely minimal. Selection bias due to low response rates and over-representation of higher educated participants likely occurred. Another selection bias may be implied by the fact that deceased survivors are not represented. Notably, both male and female participants had the same age as the initial pool of eligible survivors (*p* > 0.2), but were somewhat further away from diagnosis (mean difference, 1.1 and 1.3 years for men and women, respectively, *p* < 0.001). Moreover, relatively more men with testicular cancer participated (43 vs. 36% eligible), while fewer men with other types participated (33 vs. 43% eligible; *p* = 0.002), also limiting our ability to assess effects within various diagnostic groups and leading to a heterogeneous “other” group. In women, although significant (*p* < 0.001), types of diagnosis among included vs. eligible women were quite similar with distributions differing by less than 4%. We also relied on self-report and could not objectively assess survivors’ fertility post-treatment. Due to limited treatment-related information based on registry data, our treatment intensity rating was also generic and not specific to potential damage to the gonads. Finally, cryopreservation in more recently treated AYAs deserves more attention, but our data represent the reality of many young survivors currently living and treated at times with limited cryopreservation options for female patients.

## Conclusions

Being diagnosed with cancer at AYA age can influence survivors’ reproductive goals and disrupt their timing of having children, given that they are diagnosed during their most fertile years in life. This study highlighted different determinants of cryopreservation in male survivors, and more detailed treatment data may offer additional insights. Cryopreservation in female AYAs needs additional research. Most survivors had children by the time of the study, but reproductive goals were partially influenced by cancer, particularly in women. Utilization of ART following treatment remains low, and driving factors need further examination to optimally counsel and offer cryopreservation to patients in the highest need.

## Supplementary Information

Below is the link to the electronic supplementary material.

Supplementary file1 (PDF 323 KB)

## Data Availability

The data underlying this article are available in the article and in its online supplementary material. The datasets generated and/or analyzed during the current study are available from the corresponding author on reasonable request.
