# Supplementary material for: Pre-treatment fertility preservation and post-treatment reproduction in long-term survivors of adolescent and young adult (AYA) cancer
Source: J Cancer Surviv. 2024 Feb 6;19(4):1143–54. doi: 10.1007/s11764-024-01538-x (PMC12283814; doi:10.1007/s11764-024-01538-x)
Supplement: Supplementary file 1 — Supplementary file1 (PDF 323 KB) [file 11764_2024_1538_MOESM1_ESM.pdf]

Appendix to the manuscript:

[Journal of Cancer Survivorship]

Pre-treatment fertility preservation and post-treatment reproduction in survivors of adolescent and young adult (AYA) cancer: A population-based study in the Netherlands

Vicky Lehmann, Carla Fokkema-Vlooswijk, Winette T.A. van der Graaf, Rhodé Bijlsma, Suzanne E.J. Kaal, Jan Martijn Kerst, Jacqueline M. Tromp, Monique E.M.M. Bos, Tom van der Hulle, Roy I. Lalisang, Janine Nuver, Mathilde C.M. Kouwenhoven, Catharina C.M. Beerendonk, Christianne Lok, Marij Dinkelman-Smit & Olga Husson

Author of Correspondence:
Vicky Lehmann, PhD
Medical Psychology, Amsterdam University Medical Center, Meibergdreef 9, 1105 AZ Amsterdam
Email: v.lehmann@amsterdamumc.nl

<u>Overview:</u>

Appendix-A:
English translation of SURVAYA items and coding of outcomes for the presented analyses
[the Dutch version of all items is available upon request]

Fertility status coding: Beliefs that treatment had affected survivors' fertility

Appendix-1:
Descriptive statistics of disease stage and treatment modalities for all **male** survivors combined and stratified by major diagnostic groups (testicular cancer, lymphoma/leukemia, other), which were further stratified by whether they had completed sperm cryopreservation (in grey)

Appendix-1-Figure:
Sperm cryopreservation rates by year of diagnosis (1999-2015) for testicular cancer survivors (*n*=626), leukemia/ lymphoma survivors (*n*=356), and 'other' cancer survivors (*n*=475)

Appendix-2:
Descriptive statistics of disease stage and treatment modalities for all **female** survivors combined and stratified by major diagnostic groups (breast cancer, leukemia/lymphoma, cervical cancer, others)

Appendix-3:
Descriptive statistics of fertility status in **female** participants aged <40 years versus 40-45years at study participation

Appendix-A: English translation of SURVAYA items and coding of outcomes for the presented analyses

Do you have children? parental status**
- ☐ No
- ☐ Yes

I discussed the topic of infertility with my doctor before I started my treatment. recall consultations
- ☐ Yes
- ☐ No

Which of the following statements applies to you?
- ☐ I have taken specific steps to reduce the risk of infertility
- ☐ I have not taken specific steps, because it was not discussed with me — cryopreservation: no attempt
- ☐ I have not taken specific steps, because I didn't want to or couldn't — cryopreservation: no attempt
- ☐ I have tried, but it did not work — cryopreservation: unsuccessful attempt

Men:

Which steps did you take to reduce the risk of infertility? cryopreservation

[may choose several options]
- ☐ Shielding testis from radiation
- ☐ Freezing sperm
- ☐ Freezing sperm [extracted] from epididymis [PESA]
- ☐ Freezing sperm [extracted] from testicles [TESE]
- ☐ Freezing sperm [extracted] through electro-ejaculation
- ☐ Other: …………………………………………………………………**

Did you have biological children following your cancer diagnosis? parental status** / ways of conceiving

[may choose several options]
- ☐ No, no desire
- ☐ No, not yet, but I want to have (additional) children in the future
- ☐ No, but we are trying
- ☐ Yes, naturally (without any other measures) — ways of conceiving
- ☐ Yes, through intra-uterine insemination (IUI) — ways of conceiving
- ☐ Yes, through a surrogate
- ☐ No, it did not work despite medical assistance
- ☐ Other: …………………………………………………………………**

Women:

Which steps did you take to reduce the risk for infertility? cryopreservation

[may choose several options]
- ☐ Ovarian transposition (out of radiation field)
- ☐ Freezing eggs
- ☐ Freezing embryo's
- ☐ Freezing ovarian tissue
- ☐ Suppressing ovarian function (hormone injections)
- ☐ Other: …………………………………………………………………**

Did you have biological children following your cancer diagnosis? parental status** / ways of conceiving

[may choose several options]
- ☐ No, no desire
- ☐ No, not yet, but I want to have (additional) children in the future
- ☐ No, but we are trying
- ☐ Yes, naturally (without any other measures)
- ☐ Yes, through IVF (in vitro fertilization) or ICSI (intracytoplasmic sperm injection)
- ☐ Yes, hormonal treatment
- ☐ Yes, through a surrogate
- ☐ No, it did not work despite medical assistance
- ☐ Other: ………………………………………………………………… **

Do you think that the cancer treatment affected your fertility? fertility status [see coding below]
- ☐ Yes, please specify : …………………………………………………………………**
- ☐ No

Did your cancer diagnosis affect your wish to have (additional) biological children? effects of cancer on reproductive goals
- ☐ Yes, due to cancer I don't want to have children anymore
- ☐ Yes, due to cancer I particularly want to have (more) children
- ☐ No, my diagnosis had no effect

*** we used any information from open-ended answers that further alluded to survivors' fertility and parental status pre vs. post treatment*

Fertility status coding:
Beliefs that treatment had affected survivors' fertility

Coded as:	Answers of male survivors included:	Answers of female survivors included:
**infertile/ sterile (i.e., unable to conceive at all or without ART)**	• double orchiectomy/ "no testicles anymore"<br>• "infertile"<br>• "not possible anymore"<br>• azoospermia/ "no semen"	• Premature ovarian insufficiency (POI)<br>• Hysterectomy (with or without oophorectomy)<br>• Double oophorectomy<br>• "infertile"<br>• Needing a surrogate<br>• Needing ICSI or IVF
**impaired/ subfertile**	• low(er) motility<br>• "bad sperm quality"<br>• single orchiectomy (often mentioned in combination with chemo)<br>• test results: impaired/ "decreased"	• Single oophorectomy<br>• (temporal) POI due to hormone replacement therapy**<br>• surgeries of the cervix
**(likely) impaired, but not tested**	• "chemo"<br>• "told to be at risk"<br>• froze semen, but not tested in survivorship*	• "told to be at risk"
**(likely) fertile**	• fertility recovered over time	• Believe there were no effects<br>• Natural conception

* fertility preservation before treatment was used as indicator that they may have been at risk and thus may experience fertility problems
** fertility is currently uncertain: it is unknown whether their fertility will recover and whether that would be on a timeline where they could still conceive (vs. natural menopause)

Appendix-1

Table 1: Descriptive statistics of disease stage and treatment modalities for all male survivors combined and stratified by major diagnostic groups (testicular cancer, lymphoma/leukemia, other), which were further stratified by whether they had completed sperm cryopreservation (in grey)

N	All male survivors 1457 (100%)	Testicular cancer 626 (43.0%)		Lymphoma/ leukemia 356 (24.4%)		Other types of cancer 475 (32.6%)	
**sperm cryopreservation** [a]	330 (22.6%)	**completed**	**not completed**	**completed**	**not completed**	**completed**	**not completed**
		177 (28.3%)	449 (71.7%)	94 (26.4%)	262 (73.6%)	59 (12.4%)	416 (87.6%)
**Disease stage** [b]							
I	544 (37.3%)	311 (49.7%)		61 (17.1%)		172 (36.2%)	
		78 (25.1%)	233 (74.9%)	21 (34.4%)	40 (65.6%)	16 (9.3%)	156 (90.7%)
II	328 (22.5%)	157 (25.1%)		95 (26.7%)		76 (16.0%)	
		54 (34.4%)	103 (65.6%)	32 (33.7%)	63 (66.3%)	8 (10.5%)	68 (89.5%)
III	274 (18.8%)	157 (25.1%)		39 (11.0%)		78 (16.4%)	
		45 (28.7%)	112 (71.3%)	10 (25.6%)	29 (74.4%)	11 (14.1%)	67 (85.9%)
IV	89 (6.1%)	-		58 (16.3%)		31 (6.5%)	
		-	-	12 (20.7%)	46 (79.3%)	1 (3.2%)	30 (96.8%)
Unknown	222 (15.2%)	1 (0.2%)		103 (28.9%)		118 (24.8%)	
**Primary treatment modalities**							
Surgery	303 (20.8%)	96 (15.3%)		1 (0.3%)		205 (43.2%)	
		25 (26.0%)	71 (74.0%)	-	1 (100%)	7 (3.4%)	198 (96.6%)
Chemotherapy	219 (15.0%)	-		190 (53.4%)		29 (6.1%)	
		-	-	45 (23.7%)	145 (76.3%)	10 (34.5%)	19 (65.5%)
Radiation	53 (3.6%)	1 (0.2%)		15 (4.2%)		37 (7.8%)	
		-	1 (100%)	1 (6.7%)	14 (93.3%)	7 (18.9%)	30 (81.1%)
Surgery + chemotherapy	383 (26.3%)	357 (57.0%)		-		26 (5.5%)	
		109 (30.5%)	248 (69.5%)	-	-	8 (30.8%)	18 (69.2%)
Surgery + radiation	270 (18.5%)	169 (27.0%)		-		101 (21.3%)	
		41 (24.3%)	128 (75.7%)	-	-	11 (10.9%)	90 (89.1%)
Chemotherapy + radiation*	169 (11.6%)	-		134 (37.6%)		35 (7.4%)	
		-	-	47 (35.1%)	87 (64.9%)	9 (25.7%)	26 (74.3%)
Surgery + chemo + radiation*	40 (2.7%)	3 (0.5%)		-		37 (7.8%)	
		2 (66.7%)	1 (33.3%)			6 (16.2%)	31 (83.8%)
Other/ missing	20 (1.4%)	-		16 (4.5%)		5 (1.1%)	
**Stem cell transplant (SCT)***	68 (4.7%)	2 (0.3%)		63 (17.7%)		3 (0.6%)	
		-	2 (100%)	13 (20.6%)	50 (79.4%)	-	3 (100%)
**Treatment intensity**							
**High intensity**	260 (17.8%)	5 (0.8%)		182 (51.1%)		73 (15.4%)	
		2 (40.0%)	3 (60.0%)	58 (31.9%)	124 (68.1%)	15 (20.5%)	58 (79.5%)
**Less/low**	1197 (82.2%)	621 (99.2%)		174 (48.9%)		402 (84.6%)	
		175 (28.2)%	446 (71.8)%	36 (20.7%)	138 (79.3%)	44 (10.9)%	358 (89.1)%
**Years since diagnosis** [c]							
5+ years (5-9 years)	484 (33.2%)	178 (28.4%)		120 (33.7%)		186 (39.2%)	
		51 (28.7%)	127 (71.3%)	32 (26.7%)	88 (73.3%)	27 (14.5%)	159 (85.5%)
10+ years (10-14 years)	495 (34.0%)	221 (35.3%)		108 (30.3%)		166 (34.9%)	
		64 (29.0%)	157 (71.0%)	34 (31.5%)	74 (68.5%)	19 (11.4%)	147 (88.6%)
15+ years (15-22 years)	478 (32.8%)	227 (36.3%)		128 (36.0%)		123 (25.9%)	
		62 (27.3%)	165 (72.7%)	28 (21.9%)	100 (78.1%)	13 (10.6%)	110 (89.4%)

Note: of all men who had unsuccessfully attempted cryopreservation, 56.9% (*n*=33/58) had testicular cancer, 27.6 % (*n*=16/58) had lymphoma/leukemia, and 15.5% (*n*=9/58) another type of diagnosis. Although within types of diagnosis, this equates to similar rates: 5.3% in testicular cancer and 4.5% in lymphoma/leukemia, versus only 1.9% of other types of diagnoses. * included in high treatment intensity
[a] 96.4% (*n*=318/330) cryopreserved fresh semen; [c] *n*=18 got additional shielding from radiation as a means to spare fertility; [b] cryopreservation rates did not significantly differ by stage within each diagnostic group: testicular cancer (χ^2=4.5, *p*=.107), leukemia/lymphoma (χ^2=3.9, *p*=.268) and others (χ^2=3.1, *p*=.372); [c] cryopreservation rates were similar over time across the whole sample (χ^2(2)=0.61; *p*=.738; see Manuscript Table 1), and within each diagnostic group: testicular cancer survivors (χ^2=0.2, *p*=.920), leukemia/lymphoma (χ^2=2.8, *p*=.248) and others (χ^2=1.3, *p*=.526); see also Figure 1 below

Appendix-1-Figure:

Sperm cryopreservation rates by year of diagnosis (1999-2015) for survivors of testicular cancer (*n*=626), leukemia/ lymphoma (*n*=356), and ‘other’ types of cancer (*n*=475)

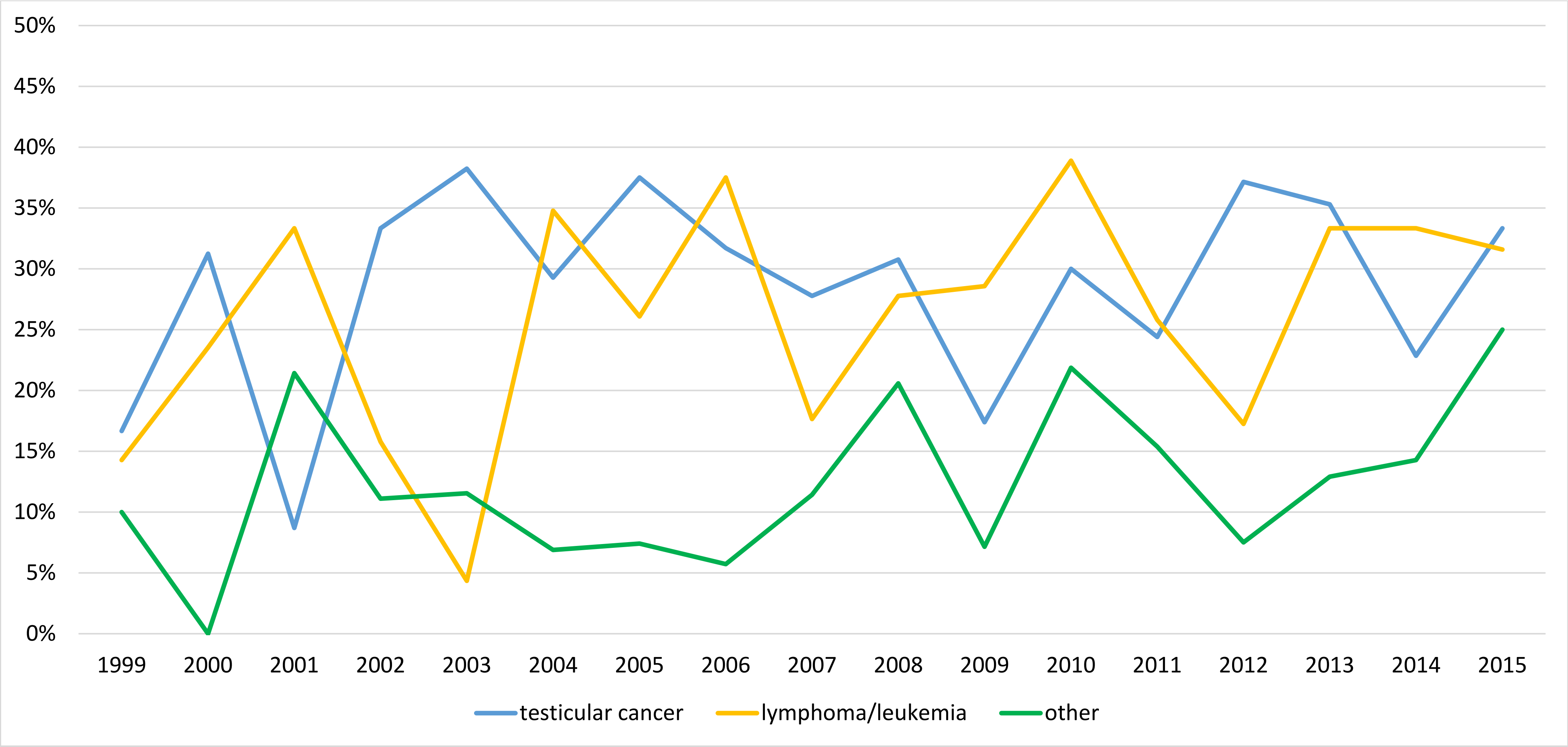

Appendix-2

Table 2: Descriptive statistics of disease stage and treatment modalities for all **female** survivors combined and stratified by major diagnostic groups (breast cancer, leukemia/lymphoma, cervical cancer, others)

	**Female survivors**	**Breast cancer**	**Lymphoma/ leukemia**	**Cervical cancer**	**other**
N	2112 (100%)	812 (38.4%)	315 (14.9%)	306 (14.5%)	679 (32.1%)
**Disease stage**					
I	992 (47.0)	299 (36.8%)	41 (13.0%)	259 (84.6%)	393 (57.9%)
II	606 (28.7%)	381 (46.9%)	102 (32.4%)	38 (12.4%)	85 (12.5%)
III	241 (11.4%)	129 (15.9%)	30 (9.5%)	1 (0.3%)	81 (11.9%)
IV	70 (3.3%)	3 (0.4%)	35 (11.1%)	6 (2.0%)	26 (3.8%)
Unknown	203 (9.6%)	-	107 (34.0%)	2 (0.7%)	94 (13.8%)
**Primary treatment modalities**					
Surgery	563 (26.7%)	37 (4.6%)	-	192 (62.7%)	333 (49.0%)
Chemotherapy	189 (8.9%)	2 (0.2%)	170 (54.0%)	1 (0.3%)	16 (2.4%)
Radiation	40 (1.9%)	1 (0.1%)	9 (2.9%)	4 (1.3%)	26 (3.8%)
Surgery + chemotherapy	206 (9.8%)	147 (18.1%)	-	9 (2.9%)	50 (7.4%)
Surgery + radiation	304 (14.4%)	85 (10.5%)	-	22 (7.2%)	197 (29.0%)
Chemotherapy + radiation*	209 (9.9%)	2 (0.2%)	127 (40.3%)	51 (16.7%)	29 (4.3%)
Surgery + chemotherapy + radiation*	586 (27.7%)	537 (66.1%)	-	27 (8.8%)	22 (3.2%)
Other/missing	15 (0.7%)	1 (0.1%)	9 (2.8%)	-	6 (0.8%)
**Stem cell transplant (SCT)***	63 (3.0%)	5 (0.6%)	55 (17.5%)	-	3 (0.4%)
**High treatment intensity**	838 (39.7%)	541 (66.6%)	167 (53.0%)	78 (25.5%)	52 (7.7%)
**Cryopreservation** [a]					
total	76 (3.6%) [b]	50 (6.2%)	10 (3.2%)	4 (1.3%)	12 (1.8%)
Oocytes	34	21	7	-	6
Embryo's	38	26	2	3	7
Ovarian tissue	12	4	1	3	4
after 2013	24/ 239 (10.0%) [c]	14/ 96 (14.6%)	5 / 42 (11.9%)	1 / 28 (3.6%)	4 / 73 (5.5%)
Oocytes	16	9	3	-	4
Embryo's	8	5	1	-	2
Ovarian tissue	3	-	1	1	1

* included in high treatment intensity

[a] *n*=11 got (additional) ovarian transportation/shielding from radiation and n=25 got hormone injections as a means to spare fertility

[b] *n*=8 indicated two options

[c] *n*=3 indicated two options

Appendix-3

Table 3: Descriptive statistics of fertility status in **female** participants aged <40 years versus 40-45years at study participation

**Perceived effects of treatment:**	**Women age <40** *n*=555	**Women age 40-45** *n*=549
No effect on fertility	256 (46.1%)	215 (39.2%)
Infertile/ sterile	95 (17.1%)	146 (26.6%)
POI [a]	*32 (33.7%)*	*38 (26.0%)*
hysterectomy [b]	*18 (18.9%)*	*37 (25.3%)*
double oophorectomy	*5 (5.3%)*	*25 (17.1%)*
unspecified [c]	*40 (42.1%)*	*46 (31.5%)*
Impaired/ subfertile	83 (15.0%)	81 (14.8%)
Likely impaired, but not formally tested	21 (3.8%)	12 (2.2%)
(likely) fertile	19 (3.4%)	13 (2.4%)
Missing	81 (14.6%)	82 (14.9%)

[a] *diagnosed with premature ovarian insufficiency*

[b] *with or without oophorectomy*

[c] *did not specify the nature of their infertility*

Note:

Natural menopause typically occurs in women between age 45-55 years, while onset between age 40-45 also occurs and is considered early menopause. Onset before age 40 is defined as premature menopause/ premature ovarian insufficiency (POI). Therefore, fertility status is reported for women aged younger than 40 years as well as 40-45 years at study participation.
